# 1038. In Vitro Activity of Tebipenem, an Orally Available Carbapenem Agent, Against a Collection of Surveillance Gram-Positive Clinical Isolates

**DOI:** 10.1093/ofid/ofab466.1232

**Published:** 2021-12-04

**Authors:** S J Ryan Arends, Abby L Klauer, Nicole Cotroneo, Ian A Critchley, Rodrigo E Mendes

**Affiliations:** 1 JMI Laboratories, North Liberty, Iowa; 2 Spero Therapeutics

## Abstract

**Background:**

Tebipenem, an orally bioavailable carbapenem administered as a pro-drug, completed a phase 3 clinical trial for evaluating its safety and efficacy for the treatment of complicated urinary tract infection and acute pyelonephritis. The purpose of this study was to investigate the *in vitro* activity of tebipenem and comparator agents, including ertapenem and meropenem, against a recent collection of Gram-positive isolates associated with clinical infections.

**Methods:**

The susceptibility of 580 Gram-positive organisms were tested, including: methicillin-susceptible *Staphylococcus aureus* (MSSA, 489 isolates), methicillin-susceptible *Staphylococcus epidermidis* (MSSE, 31), other methicillin-susceptible coagulase-negative staphylococci (MSCoNS, 29), and vancomycin-susceptible *Enterococcus faecalis* (31). The isolates were collected primarily from pneumonia in hospitalized patients (498 isolates; 85.9%), urinary tract infections (42 isolates; 7.2%), and bloodstream infections (38 isolates; 6.6%). Organisms were tested using reference broth microdilution methods in a central laboratory.

**Results:**

Tebipenem had an MIC_90_ value of 0.03 mg/L against MSSA and 0.015 mg/L against MSSE isolates. Ertapenem MIC_90_ values were 8-fold higher against MSSA (MIC_90_, 0.25 mg/L) and 32-fold higher against MSSE (MIC_90_, 0.5 mg/L). Tebipenem displayed an MIC_90_ value of 0.03 mg/L against MSCoNS species other than *S. epidermidis*. This result was 8- and 32-fold lower than those of meropenem (MIC_90_, 0.25 mg/L) and ertapenem (MIC_90_, 1 mg/L), respectively. Tebipenem inhibited all *E. faecalis* isolates at ≤1 mg/L (MIC_90_, 1 mg/L), with an MIC_90_ value at least 2-fold lower than meropenem (MIC_90_, >1 mg/L) and 16-fold lower than ertapenem (MIC_90_, >8 mg/L).

**Conclusion:**

Tebipenem displayed potent activity against methicillin susceptible staphylococci, including MSSA, MSSE, and other MSCoNS. Tebipenem *in vitro* activity was greater than meropenem and ertapenem when tested against *E. faecalis.* These data indicate that tebipenem may be an option for treating urinary tract infections caused by these organisms or as an empiric option to provide broader coverage against Gram-negative and -positive organisms.

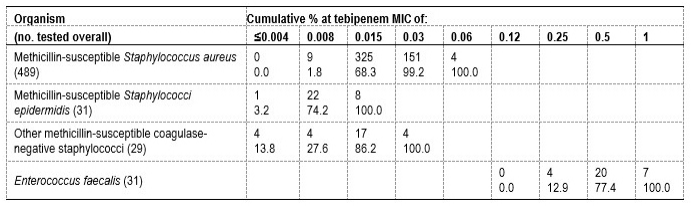

**Disclosures:**

**S J Ryan Arends, PhD**, **AbbVie (formerly Allergan**) (Research Grant or Support)**GlaxoSmithKline, LLC** (Research Grant or Support)**Melinta Therapeutics, LLC** (Research Grant or Support)**Nabriva Therapeutics** (Research Grant or Support)**Spero Therapeutics** (Research Grant or Support) **Abby L. Klauer, n/a**, **Cidara Therapeutics, Inc.** (Research Grant or Support)**Spero Therapeutics** (Research Grant or Support) **Nicole Cotroneo**, **Spero Therapeutics** (Employee, Shareholder) **Ian A. Critchley, Ph.D.**, **Spero Therapeutics** (Employee, Shareholder) **Rodrigo E. Mendes, PhD**, **AbbVie** (Research Grant or Support)**AbbVie (formerly Allergan**) (Research Grant or Support)**Cipla Therapeutics** (Research Grant or Support)**Cipla USA Inc.** (Research Grant or Support)**ContraFect Corporation** (Research Grant or Support)**GlaxoSmithKline, LLC** (Research Grant or Support)**Melinta Therapeutics, Inc.** (Research Grant or Support)**Melinta Therapeutics, LLC** (Research Grant or Support)**Nabriva Therapeutics** (Research Grant or Support)**Pfizer, Inc.** (Research Grant or Support)**Shionogi** (Research Grant or Support)**Spero Therapeutics** (Research Grant or Support)

